# Measuring the menu, not the food: “psychometric” data may instead measure “lingometrics” (and miss its greatest potential)

**DOI:** 10.3389/fpsyg.2024.1308098

**Published:** 2024-03-21

**Authors:** Jan Ketil Arnulf, Ulf Henning Olsson, Kim Nimon

**Affiliations:** ^1^BI Norwegian Business School, Oslo, Norway; ^2^Department of Human Resource Development, University of Texas at Tyler, Tyler, TX, United States

**Keywords:** semantic algorithms, semantic networks, nomological networks, latent constructs, natural language processing, measurement, organizational behavior, cross-cultural psychology

## Abstract

This is a review of a range of empirical studies that use digital text algorithms to predict and model response patterns from humans to Likert-scale items, using texts only as inputs. The studies show that statistics used in construct validation is predictable on sample and individual levels, that this happens across languages and cultures, and that the relationship between variables are often semantic instead of empirical. That is, the relationships among variables are given a priori and evidently computable as such. We explain this by replacing the idea of “nomological networks” with “semantic networks” to designate computable relationships between abstract concepts. Understanding constructs as nodes in semantic networks makes it clear why psychological research has produced constant average explained variance at 42% since 1956. Together, these findings shed new light on the formidable capability of human minds to operate with fast and intersubjectively similar semantic processing. Our review identifies a categorical error present in much psychological research, measuring representations instead of the purportedly represented. We discuss how this has grave consequences for the empirical truth in research using traditional psychometric methods.

## Introduction

This is a conceptual interpretation and synthesis of empirical studies using semantic algorithms that are capable of predicting psychological research findings *a priori*, in particular survey statistics. The main motive for this study is to sum up findings from a decade of psychological research using text algorithms as tools. As will be shown, outputs from this methodology are now quickly increasing with the advent of powerful and accessible technologies. Available research so far indicates that the phenomenon which Cronbach and Meehl (1955) described as a “nomological network” may, more often than not be of semantic instead of nomological nature. We believe that this confusion has led to decades of categorical mistakes regarding psychological measurement: What has been measured is the systematic representations of abstract propositions in the minds of researchers and subjects, not the purported lawful relationships between independently existing phenomena, i.e., the supposed contents of the construct. Hence the title of this study: measuring the “menu,” the semantic representation, instead of the “food,” the subject matter of the representations.

This proposition builds on a set of empirical evidence made possible in recent years through the advancement of natural language processing (NLP) technologies. This evidence will be presented thoroughly in later sections, but we will first give the reader a very brief introduction to the technology and why it matters for social science research. The most famous example of NLP technologies in recent years has been large language models (LLMs) like OpenAI’s “ChatGPT” or Google’s “Bard.” These tools can read inputs in natural language, discuss with human users, and produce texts that are logically coherent to the extent that they can write computer code and analyze philosophical topics.

Users who simply “talk” with the LLMs only meet their human-like responses. They do not have access to the computational workings behind the interface. However, these features are made possible through previous developments in assessing and computing semantic structures in human language. Building on decades of research, NLP approaches have found ways to represent meaning in texts by quantifying linguistic phenomena such as words, sentences and propositions (Dennis et al., 2013). Increasingly, the semantic processing techniques have been found to match or emulate similar processes in humans, narrowing the gap between human and computer capabilities (cfr. Arnulf et al., 2021).

Of particular relevance to the present topic, NLP techniques such as Latent Semantic Analysis (Dumais et al., 1988), Word2Vec (Mikolov et al., 2013), and BERT (Devlin et al., 2018) have been available to measure and compute the semantic structures of research instruments as well as theoretical models and research findings. Without going into details at this point, the mentioned technologies allow us to compute the degree to which variables overlap in meaning (Larsen and Bong, 2016). This has opened a completely new perspective on methodology because it appeared that a vast range of research findings hitherto seen as empirical were instead following from the semantic dependencies between the variables: semantic algorithms can actually predict 80–90% of human response patterns *a priori* based only on the questionnaire texts as inputs, sometimes replicating all information used to validate constructs (Arnulf et al., 2014; Nimon et al., 2016; Gefen and Larsen, 2017; Shuck et al., 2017; Kjell et al., 2019; Rosenbusch et al., 2020; Larsen et al., 2023).

It is important to understand that NLP technologies do not only map and compute wordings of questionnaires, but their calculations also pervade definitions of variables and constructs (Fyffe et al., 2023; Larsen et al., 2023). Since these calculations span the scientific process from empirically collected respondent data to the theoretical argumentation of the researchers, we need to reconsider the distinction between empirical and semantic features of data. The empirical studies to be reviewed here only come about because abstract propositions in the human mind have systematic properties that render them accessible to statistical modelling from text alone. The outline of the present study is as follows:

We will first describe how language processing algorithms can allow *a priori* predictions of response patterns to prevalent, state-of the art measurement instruments in organizational psychology (Arnulf et al., 2018a,b,c,d). Next, we will show how the prediction works across languages and culturally diverse samples (Arnulf and Larsen, 2018). We then use these research findings to re-interpret Cronbach and Meehl’s (1955) original concept of “nomological networks” with the more accurate terminology “semantic networks.” We argue that many psychological variables do not really “predict” each other in a causal or temporal sense. Instead, they are better understood as re-interpretations of each other as nodes in semantic networks. It is this feature that keeps producing construct identity fallacies (Larsen and Bong, 2016), also called the “jingle/jangle problem.”

One peculiar consequence is the empirical demonstration that construct validation conventions tend to lock the explained variance in psychological studies at a constant average of 42% (Smedslund et al., 2022). Another consequence is that semantic networks cannot express empirical truth values (Arnulf et al., 2018a,b,c,d). Semantic networks are prerequisites for the human talent to create arguments and counterfactual hypotheses (Pearl, 2009). This is precisely the reason why we have empirical science, as we need other types of information to falsify hypotheses (Russell, 1918/2007).

**Figure 1:**
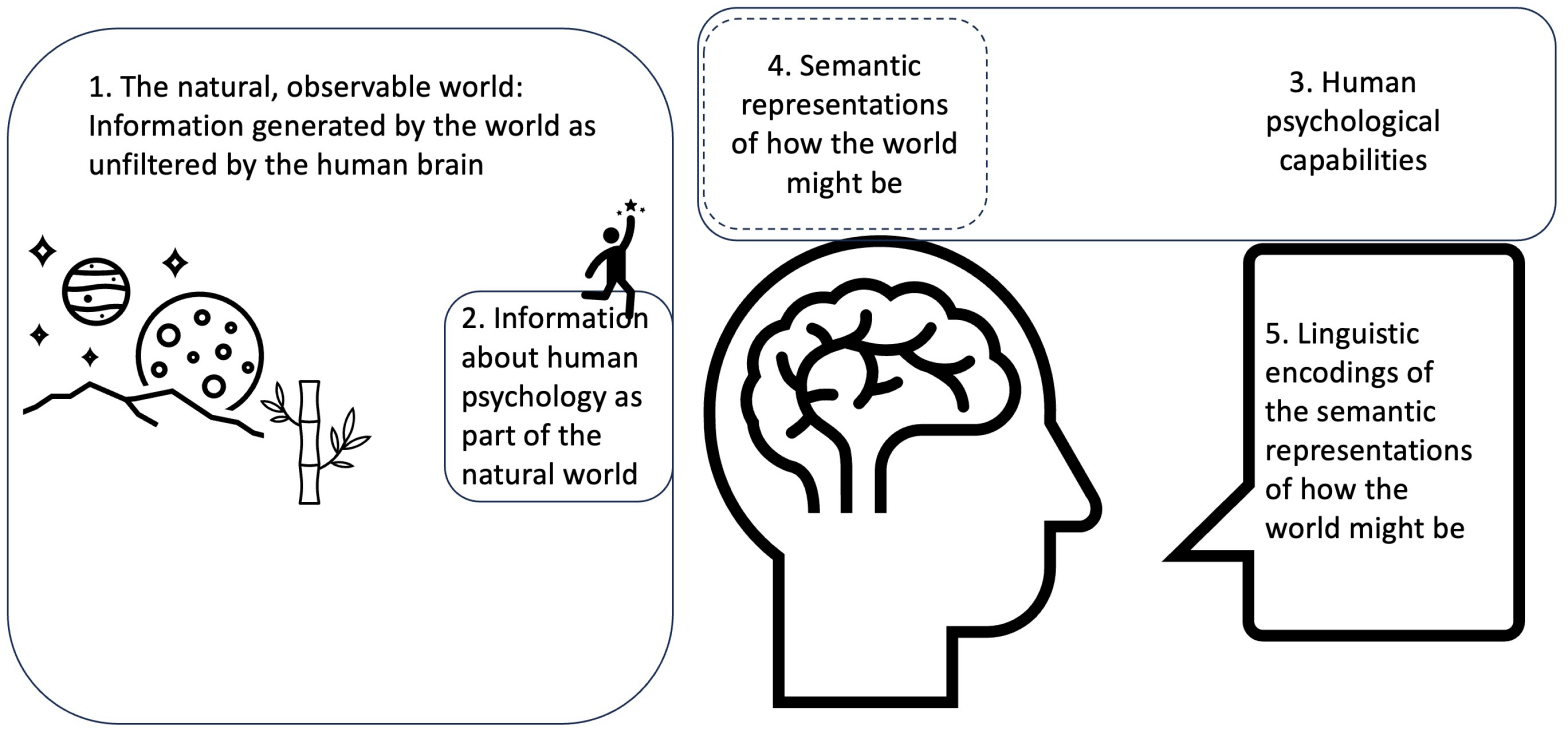
Empirical science should ideally tap into the features of the world that we cannot see (1). Psychology takes aim at a piece of this (2), but often stops at the doorstep by collecting information about how we represent the world (4). This is itself a product of our psychological apparatus (3) and we need it to talk about what we see and find (5), but it is not itself empirical certainty about 1 and 2.

Finally, we will point at possible ways to advance from here. Humans display an ability for semantic parsing that is predictable on a level unsurpassed in experimental psychology (Michell, 1994). We posit that statistic modelling of semantic processes is a necessary step to understand that psychological research is itself a revealing psychological phenomenon. The phenomena that will be addressed and discussed in this article are outlined in Figure 1.

## Prediction of empirical statistics *a priori*

Probably the most common approach to empirical psychology is to establish a theoretical relationship between two or more defined constructs, operationalize the constructs as variables and collect some types of data (Bagozzi and Edwards, 1998; Nunally and Bernstein, 2007; Borsboom, 2008; Bagozzi, 2011; Michell, 2013; Vessonen, 2019; Uher, 2021b). The testing of the hypotheses, and hence the theories, hinges in the measurement data fitting the predictions, that in turn belong to the argued theories (Popper and Miller, 1983; Jöreskog, 1993). The purpose is to allow a quantitative description of the relationship between the variables, based on the numbers obtained as measurements.

Following predominant philosophy of science, the assumption is that reasonably argued theoretical relationships should withstand attempts to falsify them (Popper, 1959). The falsification could take two steps: First, a statistical rejection of null hypotheses showing that the numbers are reasonably non-coincidental, and secondarily the hypotheses are supported (by not being disconfirmed).

Hence, psychological research abounds with complex and elaborate statistical models that either stepwise or in one sweep take all these concerns into consideration (Lamiell, 2013). If the numbers fit the statisticians’ model requirements, the findings are generally accepted as “empirically supported.” What this should imply, is that the measurement results came about as independent observations from the theoretical propositions.

A number of research traditions have over the years voiced doubt about this independence. The doubt has largely taken two forms. The first type of doubt in the data independence came from criticism of the widespread use of quantitative self-report instruments. Starting already upon Rensis Likert’s adoption of quantitative response categories to questionnaires in 1932, other researchers were concerned about the nature of the ensuing numbers as well as about the value of self-reported responses across many domains of inquiry (LaPiere, 1934; Drasgow et al., 2015).

The second type of doubt has targeted a broader and more conceptual side to psychological research, regardless of the method applied. What if the empirical data collection is set up to replicate something that is necessarily true? Many such situations are conceivable, such as finding out whether people who experience something unexpected will turn out to be surprised (Semin, 1989; Smedslund, 1995). While some such examples may be blatantly obvious, incisive theoretical analyses have found several instances of more indirect versions of this where the dependent and independent variables are found to be parts of each other’s definitions (van Knippenberg and Sitkin, 2013). Where variables are conceptually overlapping, they will also be statistically related if measured independently.

Both of these concerns allow us to state a very precise prediction: When research instruments or designs ask questions where the answers are given by the meaning of the questions used, the resulting statistics should be explainable by the texts. More precisely, the information contained in the definitions of constructs, variables and research questions comes back as the observed statistics (Landauer, 2007). When this happens, the measures are not independent information that fit the theories. The measures are measuring the semantic properties of theoretical statements in a self-perpetuating loop.

A simple example may illustrate how semantic algorithms can model empirical data. Assume that we are asking respondents about the condition of their lawn, rating the item: “The lawn is wet.” To “predict” this variable, we ask people to rate three other variables: (1) “It is raining,” (2) “It is snowing,” and (3) “the sun is shining.” We can run this example with LSA at the openly available website http://wordvec.colorado.edu/, and the results are displayed in Table 1. A mere semantic analysis of the statements is predicting the likely outcome of this empirical exercise: If it rains, the lawn is likely to be wet. By snow it is almost as likely, but if the sun shines, it is less likely to be wet.

**Table 1:** Semantic similarities between four statements about gardening.

	It is raining	It is snowing	The sun is shining	The lawn is wet
It is raining	1			
It is snowing	0.93	1		
The sun is shining	0.43	0.41	1	
The lawn is wet	**0.80**	**0.73**	**0.44**	1

Relationships with dependent variable in bold.

The important point here is not the absolute values, but the mutual quantitative relationships between variables. These semantic values can be compared to correlations or covariances, but they are not meaningful as single data points, only as relationships. The results in Table 1 are blatantly obvious but the same principles hold across far less obvious data structures.

At the moment of writing, studies demonstrating semantically predictable research findings and picking up at an increasing pace covering state-of-the-art research instruments in leadership and motivation (Arnulf et al., 2014), engagement, job-satisfaction and well-being (Nimon et al., 2016), the technology acceptance model (Gefen and Larsen, 2017), job analysis (Kobayashi et al., 2018), personality scale construction (Abdurahman et al., 2023; Fyffe et al., 2023), entrepreneurship (Freiberg and Matz, 2023) personality and mental health (Kjell K. et al., 2021; Kjell O. et al., 2021) or even near-death-experiences (Lange et al., 2015). Overlapping meanings between a vast group of constructs have been demonstrated (Larsen and Bong, 2016) and new scales can be checked for overlaps (Rosenbusch et al., 2020; Nimon, 2021).

Some of these studies will be explained in more detail below, but we first need to recapitulate some of the features of latent constructs that allow such predictions from the measurement texts alone.

## The latent construct and its cognitive counterpart

Up until the mid-1950s, mainstream psychological research was dominated by a behaviorist and/or positivist view on what constituted legitimate empirical variables (Hergenhahn, 2009). Invisible, inferred psychological phenomena like thinking and emotions were regarded with theoretical suspicion as they could not be observed directly. This changed considerably with the “cognitive revolution” that in many ways paralleled the growing understanding of information and communication theories (Shannon and Weaver, 1949; Pierce, 1980). Borrowing from the idea of “operationalism” in physics, psychology gradually warmed up to the idea of studying phenomena inside the organism by adopting “hidden” variables or through lines of argumentation that would result in “constructed” variables (Bridgman, 1927; Boring, 1945). One milestone came with Cronbach & Meehl’s paper on the statistical criteria for “construct validation” (Cronbach and Meehl, 1955). This contribution was to become the cornerstone of APA’s test manual guideline for construct validation, as the latent variable became an established feature of empirical psychology (APA, 2009; Slaney, 2017b).

Acceptance in mainstream methodology notwithstanding, latent variables still have the peculiar feature that they cannot be observed. They will always have to be inferred from operationalizations, i.e., other more empirically accessible observations that point towards the existence of a common factor. Moreover, their ontological status has never been settled within the psychological sciences (Lovasz and Slaney, 2013; Slaney, 2017a). With the advent of desktop computing in the 1980s, factor analysis became a tool for everyone and methods for modelling these proliferated (Andrich, 1996). The proliferation of statistical methods brought about a similar proliferation of new latent constructs (Lamiell, 2013; Larsen et al., 2013). Such rapid increase in constructs raised another hundred year old problem in psychological theorizing (Thorndike, 1904): How and when do we know if two theoretical variables are the same, even if they carry the same name? Or how can we know that two groups of researchers are really working on the same problem, simply by knowing the name of the construct they are working on?

This question has been named the “construct identity problem” and points to a problematic but interesting feature of human cognition that also affects researchers (Larsen and Bong, 2016): What’s in a name? It is obviously possible for humans to believe that two statements are distinct, even though they are making the same point. The all-too-human confusion on this point is a major feature in the research on decision making such as the seminal research of Kahneman and Tversky on framing (e.g., Tversky and Kahneman, 1974; Kahneman and Tversky, 1982). Recent research indicates that such problems, often referred to as the “jingle-jangle”-problem, are very real phenomena in psychological research (Nimon et al., 2016). While digital text algorithms can detect and differentiate construct identity fallacies across large swaths of constructs, humans have in fact a hard time detecting such similarities (Larsen and Bong, 2016).

Thus, the latent and elusive nature of constructs go together with a cognitive handicap in humans, the fact that we are often oblivious about overlaps and relationships between the constructs. This renders psychology and related disciplines vulnerable to linguistic fallacies since most latent variables shaping research are also everyday concepts that are known and taken for granted by most people (Smedslund, 1994, 1995). Psychological research is concerned with learning, thinking, emotions, perception and (mostly) easily understandable constructs in healthy and disturbed personalities (Haeffel, 2022). But can we be certain that everyday concepts can be treated as fundamental entities of psychological theory – latent variables – just because their measurement statistics correspond to APA requirements from 1955 (APA, 2009)?

Or is it time to move on, to see that we have been doing research on questions that were largely determined – and in fact answered – by our own cognitive machinery? What would psychology look like if it could peek beyond the “manifest image” of the latent constructs and the computational machinery that makes us construct them (Dennett, 2013; Dennett, 2018)? We will now turn to discuss the empirical findings that could help us find such a perspective.

## Predicting leadership constructs

By 2014, the world’s most frequently used questionnaires on leadership was the Multifactor Leadership Questionnaire (MLQ, Avolio et al., 1995), figuring in more than 16,000 hits on Google Scholar. A study published that year (Arnulf et al., 2014) showed that the major parts of factor structures and construct relationships in the MLQ was predictable through text algorithms, using only the item texts as inputs. By running all the questions (or items) of the questionnaire through Latent Semantic Analysis (LSA) (Dennis et al., 2013) similar to the procedure in Table 1, it was possible to calculate the overlap in meaning among all items involved. The LSA output matched the observed correlations almost perfectly. Depending on the assumptions in the mathematical models, it was possible predict around 80 to 90% of the response patterns of humans from semantic similarities (Arnulf et al., 2014).

## Individual response patterns

Given the possibility that sample characteristics are predictable *a priori*, does this also apply to individual response patterns? Semantic predictions cannot know which score level a given respondent will choose when starting to fill out the survey. But, since all items are linked in various ways to all other items (weakly or strongly), it should theoretically be possible to infer something about subsequent responses after reading a few initial ones? Another study addressing precisely this question discovered that knowing the first 4–5 items of the MLQ allowed a fairly precise prediction of the 40 next responses (Arnulf et al., 2018b). In other words, the semantic relationships are not restricted to samples, they emerge already as features of individual responses. This amazing semantic precision was already predicted by unfolding theory in the 1960s (Coombs and Kao, 1960).

## Human linguistic predictability

Reading and parsing sentences comes so easily to people that it feels like reacting directly to reality. And yet, tasks like reading, comprehension, and responding to survey items are all behavioral processes based on psycholinguistic mechanisms in the brain (e.g., Poeppel et al., 2012; Krakauer et al., 2017; Proix et al., 2021). The first central feature of the semantic processing is remarkable but easily overlooked: It provides a rule-oriented predictability to people’s verbal behavior unlike any other behavior systems known in psychology except biological features of the nervous system, allowing humans to easily parse and rank texts like survey items along their semantic differentials (Michell, 1994).

Therefore, a semantic representation of Likert-scale survey items may allow us to predict the statistical patterns from both samples and individuals. Since these levels of predictability exceed most other processes in psychology (Michell, 1994; Smedslund et al., 2022), it is highly likely that semantic similarity numbers are matching and quite probably mirroring the outputs of the linguistic processes of the brain itself (cfr. Landauer, 2007). However, the process must take place on the semantic levels, not the basic linguistic parsing. The cognitive features of constructs seem relatively independent of the words used to encode them. We will show this by showing how semantic algorithms can model constructs across cultures and languages.

## The cultural invariance of semantic relationships

The study on semantic features of the MLQ described above (Arnulf et al., 2014) had an interesting design feature: The algorithm predicting the numbers worked on English language items as inputs and was situated in Boulder, Colorado while the respondents filling out the survey were Norwegians, filling out a Norwegian version of the MLQ. The algorithm knew nothing about Norwegian language or respondents. While previous research had established that LSA could work across languages (Deerwester et al., 1990), it was not obvious that propositional structures in research topics such as leadership would be statistically similar across linguistic lines. It turns out to be possible to demonstrate this similarity across even greater divides.

One study was designed to demonstrate how propositions about leadership appear as universally constant across some of the biggest linguistic and cultural divides that exist (Arnulf and Larsen, 2020). The method was applied to a very diverse group of respondents from China, Pakistan, India, Germany, Norway, and native English speakers from various parts of the world. The non-English speakers were divided into two equal groups, one responding in their mother tongue, the other half responding in English. Again, the semantics were calculated with LSA, using English language items only.

For practical purposes, the LSA output performed completely unperturbed by the linguistic differences. As in the first study, the LSA numbers almost perfectly predicted the response patterns of all groups that responded to items in English. The groups responding in other languages were slightly less well predicted and one might have speculated that there were “cultural” differences after all. However, the methodological design allowed comparisons of all groups responding in either English or their mother languages and they did not share any unique variation. In other words, there were no commonalities attributable to culture or other group characteristics. The differences in predictability across these experimental groups could only be explained by imprecise translations of the items. The propositional structures of the original instrument were picked up and used uniformly across all respondents. In other words, the propositions can be modelled statistically independently of the language used to encode them.

Both algorithms and human respondents reproduce the relationships of abstract meaning among the items. In that sense, the patterns are abstract transitive representations of the sort that “If you agree to A, you should also agree to B, but disagree to C…” as predicted by unfolding theory (Coombs and Kao, 1960; Coombs, 1964; Michell, 1994; Kyngdon, 2006). The culturally invariant feature of semantics is very important because it shows that semantic networks are prerequisite for language, but not language itself. The system of propositions hold in any language, including sign language (Poeppel et al., 2012). This is the whole point of accurate translations and back-translations in cross-cultural use of measurement scales (Behr et al., 2016). We posit that the data matrices from humans in any language match that of the LSA algorithm not because any language is correct, but *because their deeper semantic structure have identical mathematical properties* (Landauer and Littman, 1990; Landauer and Dumais, 1997; Landauer, 2007). In turn, this raises another problem because the constructs are then never truly independent – they derive their meanings from their mutual positions in the semantic network, as we will show next.

## The not so empirical variables

While “causality” is a strong word in the sciences (Pearl, 1998, 2009), most study designs in quantitative social science explore how one variable changes with changes in the another. To study an empirical relationship is usually taken to mean that the focus variables are free to vary, and that quantitative regularities between the two were unknown or at least uncertain prior to the investigation.

Explorations of the semantic relationships between variables indicate that frequently, the variables involved in psychological studies are *not* independent of each other. To the contrary, they may actually be semantic parts of each other’s definitions and belong to the same phenomenon (Semin, 1989; Smedslund, 1994, 1995; van Knippenberg and Sitkin, 2013; Arnulf et al., 2018c). To underscore this point, the above mentioned study of leadership scales found cases where the semantic algorithms predicted the relationships among *all* the involved variables (Arnulf et al., 2014). The predicted relationships were not restricted to the MLQ but spilled over into all other variables argued to be theoretically related to transformational leadership. Semantic patterns detected the relationships between independent variables (in this case, types of leadership), mediating variables (in this case, types of motivation) and dependent variables (in this case, work outcomes).

When this happens, constructs are in no way independent of each other. They are simply various ways to phrase statements about working conditions that overlap in meaning – a sort of second-order jingle/jangle relationships (Larsen and Bong, 2016; Nimon, 2021). In our example, it means that definitions and operationalizations of work imply a little bit of motivation. The definition of motivation, in its turn, implies a little bit of work effort. But the definitions of leadership and work effort show less overlap. The statistical modelling makes semantic relationships *look* like empirical relationships, where leadership seems to affect motivation, in turn affecting work outcomes. But this is just the way we talk about these phenomena, just as Thursdays need to turn into Fridays to ultimately become weekends.

## Lines of reasoning – nomological or semantic networks?

Our failure to distinguish between semantic and empirical relationships is itself an interesting and fascinating psychological phenomenon. When we are faced with a line of reasoning, it may seem intuitively appealing to us. Our need for empirical testing stems precisely from the fact that not everything that is arguable is also true as a fact. Counterfactual thinking is crucial to human reasoning (Pearl, 2009; Pearl and Mackenzie, 2018; Mercier and Sperber, 2019). But, conversely, some of the facts we find are probably true simply because they are arguable – they are related in semantic networks (cfr. Lovasz and Slaney, 2013).

This crucial point was raised by Cronbach and Meehl in their seminal paper that founded the psychometric tradition of construct validation (Cronbach and Meehl, 1955). We will show how the semantic properties of constructs can be explained by a re-interpretation of Cronbach and Meehl’s “nomological network,” spelled out as six principles and explained over two pages (Cronbach and Meehl, 1955, pp. 290–291).

A bit abbreviated, the six principles state that: (1) A construct is defined by “the laws in which it occurs.” (2) These laws relate observable quantities and theoretical constructs to each other in statistical or deterministic ways. (3) The laws must involve observables and permit predictions about events. (4) Scientific progress, or “learning more about” a construct consists of elaborating its nomological relationships, or of increasing its definite properties. (5) Theory building improves when adding a construct or a relation either generates new empirical observations or if it creates parsimony by reducing the necessary number of nomological components. (6) Different measurement operations “overlap” or “measure the same thing” if their positions in the nomological net tie them to the same construct variable.

We propose that these six principles do not spell out an empirical nomological network. The word “nomological” as invented by Cronbach and Meehl means “governed by laws” (from the Greek word “nomos” meaning law) and would imply that there are lawful regularities between the constructs. However, causal laws are never described between psychological constructs – they are always modelled as correlations or co-variances. In fact, at the time the “nomological networks” were proposed, psychological statistics was ideologically opposed to laws and causation under the influence of Karl Pearson (Pearson, 1895, 1897; Pearl and Mackenzie, 2018). Instead, the six principles outlined by Cronbach and Meehl perfectly describe the properties of a semantic network, where all nodes in the network are determined by their relationships to each other (Borge-Holthoefer and Arenas, 2010).

Here follows our re-interpretation of Cronbach & Meehl’s six principles as semantic networks (principles annotated with an “S” for semantics):

(S1) A construct is defined by “the semantic relationships that define it.” (S2) These semantic relationships explicate how the construct is expressed in language, and how it may be explained by other statements. (S3) The relationships must involve concrete instances of the constructs linking them to observable phenomena. (S4) Scientific progress, or “learning more about” a construct consists of expanding its semantic relationships, or of detailing its various meanings. (S5) Theory building improves when adding another construct can be argued to expand the use of the construct, or if it creates parsimony by reducing the number of words that we need to discuss it. (S6) Different measurement operations “overlap” or “measure the same thing” if their positions in the nomological net tie them to the same construct variable.

Next, we will show how the semantic network works in theory and research on the construct “leadership,” using a commonly used definition provided by Northouse (2021, p. 5):

(E1) “Leadership is a process whereby an individual influences a group of individuals to achieve a common goal.” (E2) This implies a series of interactions between one human being and a group of others, where the first individual has a wide range of possible ways to influence cooperation in the group of others. (E3) There must be some form of communication between the leading individual and the others, as well as involving a time dimension that allows a process to take place. (E4) Scientific progress may consist of explicating what “influence” may mean, and also about what a “process” might be. (E5) Theory building improves if other concepts such as “motivation” can expand the use of the construct, or if it creates parsimony by explaining what the group will be doing instead of having to describe the behaviors of all group members in detail. (E6) The definition covers agency in the form of influence, groups of individuals, time lapse (process) and end states (goals). Other ways of describing the process may overlap if capturing agency, influence, groups, time laps and end states in different ways.

The two ideas of nomological vs. semantic networks are strikingly similar but have very different implications. Semantic networks do not require any other “laws” than precisely a quantitative estimate of overlap in meaning. In parallel, prevalent techniques for construct validation never require data that go beyond correlations or covariations (Mac Kenzie et al., 2011). Our main proposition is that if semantic structures, obtainable *a priori*, allow predictions of the observed relationships between variables, then the network properties are probably rather semantic than nomological.

This distinction between nomological and semantic networks is crucial to understand the true power of semantic algorithmic calculations. The semantic networks between concepts (or, for that matter, “constructs”) are what allows us to reason and argue (Mercier and Sperber, 2019). It is precisely this feature of concepts that make up the logical argumentation in the “theory” part of our scientific productions. Moreover, the very idea about “constructs” that Cronbach developed was taken from Bertrand Russell’s argument that one should be able to treat phenomena as real and subject them to science if they can be inferred logically from other propositions (Slaney and Racine, 2013).

At the same time, the fact that there exists a semantic network that we ourselves easily mistake for a nomological one reveals a very intriguing feature of human psychology: Our abstract, conceptual thinking is following a mathematically determined pattern, but we ourselves are not aware of it (Dennett, 2012). The semantic algorithms allow us to model structures of abstract propositions simply from the properties of sentences – which is most likely what the human brain itself is doing (Landauer and Dumais, 1997; Landauer, 2007).

Oddly, what this implies is that much of the assumedly empirical research implying construct validation is not actually empirical (Smedslund, 1995, 2012). Instead, this type of research is more akin to a group-sourcing theoretical endeavor, establishing what can reasonably be said about constructs in a defined population of respondents. Hence the title of this article: The measurements collected are not measuring what the scales are “about.” Instead, the measurements are measuring what we are *saying* about these things. It is a mistake of categories, mistaking the menu for the food or the map for the terrain (Russell, 1919; Ryle, 1937).

The good thing about this type of research though is that it can be seen as a theoretical exercise, establishing that the theoretical relationships between items make sense to most people. For example, the cross-cultural research on transformational leadership that claims to find similar factor structures across cultures tells us only that all people agree how statements about leadership hang together. This is far from establishing contact with behavior on the ground, but it is precisely the essence of a theoretical statement.

If constructs, or the semantic concepts that make them up, have reasonably stable properties that define them, then there must be deterministic procedures in the brain to evaluate the overlap in meaning of statements. Whether software or wetware arrive at these evaluations in the same way is not important. The important part is that the coherence of statements is a mathematically representable structure, like Landauer has shown for LSA (2007) or Shannon has shown to be the case for general information systems (Shannon and Weaver, 1949; Pierce, 1980).

Due to this, it is possible to mutate constructs into propositions that overlap in meaning despite being encoded in different words. In the early years of analytical philosophy, the German logician Gottlob Frege was able to show this (Frege, 1918; Blanchette, 2012). He made a crucial distinction between “reference” and “meaning,” a precursor to the jingle/jangle-conundrum: Different sentences may refer to the same propositional facts even if they have no words in common. Their meaning however can be slightly different, capturing many layers of linguistic complexity.

In this way, human subjects often miss how data collection designs simply replicate the calculations in the semantic network. What we see here is the constructive feature of our semantic networks: They allow systematic permutations of all statements that can be turned into latent constructs precisely because they have systematic features.

## The 42% solution

Interestingly, it turns out that when constructs share less than 42% of their meaning, humans experience them semantically separate, and therefore possible targets of empirical research. Evidence for this has been found in a study that analyzed a wide range of constructs and across psychological research (Smedslund et al., 2022).

This study reviewed all the publications listed in the PsycLit database (and that referred to explained variation in the abstract) and found that the average explained variation every year since 1956 was exactly 42%. This number kept being remarkably constant throughout seven decades and 1,565 studies, including both self-report and independently observed data. By reconstructing 50 of them with only semantic data, it became evident why the number has to approximate 42. This is simply the average percentage of semantic overlap between any construct and its neighboring constructs in studies where the constructs are separated by factor analysis. In this way, psychological method conventions have built a scaffolding around our conscious experience of semantic similarities.

One may think of the mechanism this way:

Variables – or measurement items – with obvious overlap in meaning will always be grouped under the same construct. So, in factor analysis, such highly overlapping clusters will emerge as single factors. In the same way, other factors that are included in the analysis will have to appear with much smaller cross-loadings. Different schools of methodology have different cutoffs here, but usual benchmarks are minimum 0.70 for within-factor loadings and maximum 0.30 for cross-loadings. By this type of convention, most studies will publish ratios of within-and cross-loadings around these values. If one divides cross-loadings of 0.30 with within-factor loadings of 0.70, one gets exactly 42%. In plain words, the cross-loadings consist of semantic spillovers from each construct into its neighbors, allowing on average 42% shared meaning between constructs.

The reconstruction of 50 such studies using purely semantic processing of items and/or construct definitions made it clear that the semantic structure alone will yield a mutual explained variance of around 42%. This is another indicator that Cronbach and Meehl’s network is not “nomological” but most probably semantic.

However, the most important aspect of this discovery is the implication for psychological theory and epistemology in that the explained variance between constructs is locked within two other features of semantic processing: If two variables are too semantically distant, they will rarely be of interest (cannot be argued to have a relationship), and so they will probably not be researched. Conversely, if their representations have too tight semantic connections, they will be perceived to be the same construct or at best facets of the same.

In this way, the structure of semantic relationships will prepare researchers in the social sciences to design studies in a range from a few percent to maximum 42% overlap, which is what we find to be the average case across all studies reporting percentages of explained variance in the abstract or key words. We must assume that relationships of less than 42% overlap are not immediately obvious to humans as being systematically related through semantics – but they still are.

But why should we care about the difference between a semantically constructed entity and an empirical discovery, if both discoveries seem illuminating and true to humans anyway? The answer is actually alarming – semantic networks do not care whether a calculated relationship is “true” or not. It only maps how propositions are mutually related in language. Therefore, it is definitely possible to propose falsehoods even if the propositions make sense to speakers, a key condition for undertaking scientific investigations (Russell, 1922; Wittgenstein, 1922; Pearl, 2009).

## How semantic networks are oblivious to truth values

To understand this, it is useful to think about theoretical variables from two different perspectives. From one perspective, we are interested in what variables are about (Cohen et al., 2013). Researchers may be interested in how much we like our jobs, if we are being treated fairly by our employers or whether we think politicians should spend more on schools. When humans respond during data collection, their responses are about what the questions are about (Uher, 2021b).

But from a different perspective, the researchers are often only interested in whether two or more variables are related, no matter their actual strength or value (McGrane and Maul Gevirtz, 2019). This type of relationship is what correlations and covariances are built on and are most often the focus of psychological research. Note that such numbers are only quantifying the relationship itself, but abstracted from what the variables were “about” (Lamiell, 2013; Uher, 2021a).

This problem is most prevalent in research relying on verbal surveys such as Likert-scales (but not restricted to them). Consider two different persons, having different opinions on two questions. One person is giving off the enthusiastic responses, maybe ticking off 6 and 7 on two questions. The next respondent is negative and ticks off only 1 and 2 on the same two questions. From the point of view of their attitude strength, these two persons are clearly different and on opposite ends of the scale. But their systematic relationships with the two questions are exactly identical and they will contribute to the same group statistics and the same correlations in exactly the same way. This is of course the essence of correlations and should not matter if the numbers keep their relationships with their “measurable” substrates (Mari et al., 2017; Uher, 2021a). Looking at measurement from a semantic point of view, this does not seem to happen:

One study using semantic algorithms found a way to tease apart the attitude strength in individual human responses - what a variable is “about” – and the pure relationships between the variables, regardless of their contents (Arnulf et al., 2018a,b,c,d). By differing between the information about how strongly people feel about something, and the mere distance between the variables, it seemed that only the distances between scores played a role in the statistical modeling, not the absolute score levels. Most importantly, this is the part of statistical information that relates most strongly to the semantic structure of these variables. This implies that the way we model propositions semantically is independent from believing that they are true. In fact, commonly used statistical models seem to leave no information left about the topic respondents thought they were responding to. What the models contain are the mutual representations of the variables as propositions. This can be no coincidence as these structures probably mirror their mathematical representations in our cognitive apparatus.

One of the most ingenious yet least understood features of human cognitive capabilities is how we can think, formulate and communicate a near-to infinite range of propositions (Russell, 1922; Wittgenstein, 1922; Wittgenstein, 1953). The possibility to pose hypothetical, competing and counterfactual propositions is probably the very core of causality as understood by humans (Pearl, 2009; Harari, 2015). A crucial condition for “strong artificial intelligence” is arguably the implementation of computational counter-factual representations (Pearl, 2009; Pearl and Mackenzie, 2018).

Thus, while much of the semantic structures uncovered by psychological science might not tell us much about the outside world – what the constructs are “about” (the references), this discovery might actually open up another very interesting perspective. Semantic modelling may help us understand the human mind, and in particular that of the scientists themselves.

## Why algorithms perform as a one-man-band social scientist

Two recent conference papers (Pillet et al., 2022; Larsen et al., 2023) have explored this along two steps: The first step hypothesized and found that the semantic patterns can be used to determine correct operationalizations of constructs. By applying a layer of machine learning on top of the LSA procedures the algorithms could predict correctly which items belong to which construct in a sample of 858 construct-item pairs.

The next step was a test of how the algorithms do compared to humans in the item-sorting task recommended in construct validation, determining which items would make the best fit with theoretically defined constructs (Hinkin, 1998; Hinkin and Tracey, 1999; Colquitt et al., 2019). The algorithms seemed to perform as well as the average humans in deciding if items belong to constructs or not.

If we compare this with the performance in the previously cited articles, we can see that the language algorithms are able to predict data patterns that range from construct definition levels via item correspondent levels (Larsen et al., 2013; Rosenbusch et al., 2020; Nimon, 2021), down to patterns in observed statistics bearing on construct relationships and correlation patterns from human respondents (Arnulf et al., 2014; Nimon et al., 2016; Gefen and Larsen, 2017; Arnulf et al., 2018a,b,c,d).

To sum it up, the semantic algorithms seem able to predict theoretical belongingness of items, the content validity of the items, and the factor structures emerging when the scales are administered. The algorithms can predict individual responses given a few initial inputs, as well as the relationships among the latent constructs across the study design. Taken together, the algorithms seem to trace the systematic statistical representation of the whole research process – from theory to measurements, and from measured observations to variable relationships and factor loadings.

This indicates that there must exist a main matrix within which all the other definitions and measurement issues take place. The whole research process is embedded in semantic relationships from broad theoretical definitions and relationships, through the piloting efforts in sampling suitable items all the way to the final emergence of factor loadings.

This semantic matrix is the very condition for humans to communicate in language. For a word to be a meaningful concept, it needs to be explainable through other words. There is no such thing as a word in isolation. Thus, the phrase “you shall know a word by the company it keeps” actually works in the opposite direction: Words derive their meanings from being positioned relative to their neighbors (Firth, 1957; Brunila and LaViolette, 2022) in the semantic matrix of humans. All latent constructs are embedded in a calculable network which needs to have stable representations across speaking subjects.

At first glance, the requirement of stable semantic networks seems to contradict the differences between people involved in the process of generating measures and those responding to them. There are highly specialized researchers, there are purpose-sampled piloting groups in the development phase and there are the final targeted groups of respondents. There are even controversies in the literature as to whether the test samples in the piloting phase should be experts or lay people.

The semantic network does not seem to be disturbed by this in a major way. It appears so rigidly identical across humans that it feels like a manifestation of nature itself. How inter-subjectively constant is the semantic network really, and can it be computationally addressed?

## Semantic networks across respondents

The methodological gold standard of construct validation in psychology has arguably been the paper of Campbell and Fiske in 1959, claiming that only multi-trait, multi-method (MTMM) designs can estimate measurement errors to an extent that allows the true nature of a construct to be modelled across measurements (Campbell and Fiske, 1959). This is the traditional core of construct validation. By measuring a phenomenon from several angles (often referred to as “traits”) and using several methods or sources of information (referred to as “methods”) – we can see if a phenomenon has an existence relatively independent of the ways we measure it (Bagozzi and Edwards, 1998; Bagozzi, 2011).

A study building on five different datasets and involving constructs from four different leadership theories, investigated how semantic relationships appear can be modelled within a traditional MTMM framework (Martinsen et al., 2017). In one variation of this design, three different sources rated the appearance of leadership along three different facets or traits of leadership. The semantic representations of the items (generated in LSA) were added to the modelling procedure. In practice, this implied that a manager was rated by him-or herself, by a higher-level manager, and by a subordinate. The design was replicated five times with different people and different constructs. For all datasets, the semantic properties of the relationships between the measurement items were added to the model. The purpose was to establish whether the semantic representations were trait or methods effects, or if they simply captured the errors.

The numbers calculated by semantic algorithms were, in a first step, significantly correlated with the empirical covariance matrix. After fitting the MTMM model, the model implied matrix and its three components were still correlated with the semantic measures of association on a superficial level in four of the five cases.

As the analysis split the covariance components into source, method, and error, the semantic values were present in the trait components in three out of four studies but with only negligible traces in the methods components. The semantic predictability of response patterns was most clearly found in the trait components, or in other words: The validity of a latent construct is equal to its semantic representation – across all the respondents. The semantic properties *are* the construct, they are not an approximation of it.

This became distinctly clear by computing a completely new model of the data, called the restricted-error-correlation named REC-MTMM (Satorra et al., 2023). This model had a near-to perfect fit with the data. The REC-MTMM model implies that the observed sample covariances decompose as the sum four covariance elements of trait (T), source (S), REC parameter (REC), and residual (R) components. The model is accurate enough so that the residual does not contain relevant information.

*We believe that the REC-MTMM correlations and parameters are the imprints of semantic associations.* The fact that parameters can be restricted to be equal across respondents indicates that the respondents are remarkably synchronized in their way of reading the items. This holds even as they *rate* the items differently. In fact, respondents in the three different sources were only partially in agreement about the level of leadership exercised by the person they rated – their attitude strength.

Where their agreement was beyond any doubt, was in being unified in their semantic coherence with the trait characteristics. They all agreed that the items, mutually, had the same meanings. *What this implies, is that no matter how diverse the respondents’ experiences of their situations was, they would always unite in a linguistic behavior describing a possible situation.* They were in fact endorsing the properties of the semantic network, just not agreeing about the truth value of what the items proposed.

To understand the implication of this, consider a law case brought up before a court. Someone is accused of theft. All involved – the prosecutor, the defendant, and the judge – will agree that the law categories of robbery, theft or innocence exist and what they mean, but will disagree whether they actually apply in this particular case. In the same way, the respondents of our studies agreed about the various possible categories of leadership but would not always agree of the rated person was “guilty” of this type of leadership.

The REC-MTMM model is effective in bringing out the inter-subjective nature of the semantic network as a common interpretational framework for all people implied. When the fit statistics of the model are as impressive as we find here, it means that we have captured the data generating process itself. It is semantic modeling of the construct and its representation in the language of the respondents that drives this process. The salient function of the network is to provide a common conceptual framework from which speakers can communicate their assessments. Note however in line with what has been described above that the network calculations are indifferent to the truth values of the subjects as long as they can describe the situation in terms of the involved concepts or constructs.

This is the final feature of the semantic network that we want to list in this discussion of the phenomenon. It is a fundamental, intersubjective function with a predictive capability that is beyond anything else in psychology. The involved respondents have all sorts of opinions about a prevailing situation, but they all seem to agree that the situation can reliably and validly be discussed in terms of the involved constructs, as expressed in the REC-MTMM model. A model is a theory of the processes that gave rise to the data that we see, and the REC model taking semantics into consideration makes exhaustive use of all information available.

## Peeking past the semantic matrix: empirical questions

As the almost omnipresent influence of the semantic network is laid bare, one could easily wonder if almost all psychological phenomena can be predictable *a priori* through semantic relationships. What is there left to detect in terms of empirical questions?

This question is maybe one of the most pressing challenges to overcome for psychology and many other social sciences to move forward. If we keep on conducting research on relationships that are already embedded within the semantic network, we will be “addicted to constructs” (Larsen et al., 2013) forever. This practice is very similar to publishing each entry in the multiplication tables. As most children understand around the age of ten, you cannot “discover” the entries of the multiplication table – discovery is superfluous if the numbers are given by applying multiplication rules on the symbols.

One major psychological research question is to explore and describe the nature of the possible neurobiological foundations and its impact on how we represent the world. The ability of the human linguistic system to detect and encode abstract information could arguable be one of the brain’s most advanced features. The profound grasp of semantic representations that can be evoked and processed in most normal people is so precise and automated that we mostly take it for granted (Poeppel et al., 2012). The features of the semantic matrix are probably experienced like the nature of numbers, where humans have struggled for ages to determine whether the numbers are a part of nature or a human invention. In fact, most people probably have the feeling of being in direct contact with reality when coming in contact with the precise and solid patterns provided by semantics.

Yet, the semantic patterning of abstract propositions is no more a feature of nature than the longitudes and latitudes of geography. This delicate intertwining of our semantic representational system with the way that we describe and discuss the world is precisely the reason why it is so hard to discuss and grasp in our scientific findings (Russell, 1922; Wittgenstein, 1922; Mercier and Sperber, 2011). In this sense, semantic representations are to abstract thinking what Dennett (2013) has called the “manifest image” of the physical world. It is nature’s remarkably engineered cognitive illusion demanding its own empirical research field.

Another important development would be to start using the semantically calculable relations as the starting point of our scientific investigations. If psychology is to “stop winning” (Haeffel, 2022), and move towards non-obvious expansion of our knowledge, we must stop being impressed by discovering relationships that are knowable *a priori* by semantic calculations (Smedslund, 1995).

In this way the semantic matrix could pose as a Bayesian prior to research in the social sciences (Gelman and Shalizi, 2013). One can now compute the likely relationships among all variables prior to making empirical data collections (Gefen and Larsen, 2017; Arnulf et al., 2018a; Kjell et al., 2019; Gefen et al., 2020; Rosenbusch et al., 2020; Kjell K. et al., 2021; Nimon, 2021). From a statistical point of view, one should ask questions like who, how, why, and how much people will comply with what is semantically expected.

One study on motivation using semantic analysis found significant differences on individual and group levels in the way that people complied with semantic patterns (Arnulf et al., 2020). Here, different professional group made important group-level deviations from what was semantically expected in a way that correlated highly with the professions’ income levels. This calculation involved three data sources with no possible endogeneity: There were the various professions’ response patters, combined with the LSA calculated semantics, related to income levels as reported in the national statistics (not from self-report). The numbers strongly suggested that people with higher income levels and education would be directed in their ratings of motivation by a semantic grid that probably matched that of the researchers. People with lower income seemed to twist the meaning of the motivation-related items towards slightly, but significantly different meanings.

In this sense, the semantic grid is not a cast-iron structure. It is probably more like a representational capability with remarkable precision, but not without being malleable in the face of personal and cultural experience. Looking at today’s society and challenges in psychology, we are actually faced with challenges to semantic stability at a magnitude that affects political stability. Aside from our psychiatric diagnoses and clinical theories being a source of instability and conflict (Clark et al., 2017), the semantic wars seem to engulf gender, race, political belongingness and perceptions of information trustworthiness (Furnham et al., 2021; Furnham and Horne, 2022).

Given how semantic matrices supply us with experiences of conceptual reality, one should perhaps not wonder that people who are pressed towards conflicts with their own semantic structures will react emotionally, even violently, probably related to what we know about cognitive dissonance (Festinger, 1962; de Vries et al., 2015; Harmon-Jones, 2019). With increasingly powerful computational tools available, we should start describing and outlining the semantic grid to peek at what is behind the horizon.

## The possible neurobiological substrate of the semantic grid

The purpose of the present text is to argue the existence of a semantic representational system that is precise, lean, and not in itself subject to conscious observation. It is possible that this feature of verbal comprehension is founded on a neurobiological correlate. The phenomena we describe are too precise, too independent of culture and too abstract to be the result of local learning processes. Hypothesizing such a mechanism could help to understand its pervasive nature, much like color vision is thought of as a feature of the nervous system and hard to explain to the color blind.

More specifically, we hope to define and identify the mechanism here described as the “semantic grid.” Particularly relevant to this pursuit are two arguments explicated by Poeppel et al. (2012) and Krakauer et al. (2017): first, we are trying to delineate this phenomenon so precisely in terms of behavior that a neurobiological substrate could be hypothesized and tested. And second, we describe a function operating on a different level from most of the receptive and productive circuits involved in producing language behavior.

Several lines of studies indicate that the semantic coding of speech content is a specialized function separate from syntactic processing and spanning multiple words (Hagoort and Indefrey, 2014), a process separate from any sensorimotor processing of language. Semantic processing of complex propositions seems associated with a specific area of the medial prefrontal cortex (Hagoort and Indefrey, 2014, p. 354). Concomitantly, world knowledge seems to be treated differently from semantic knowledge in the brain. And finally, the parsing of a literal sentence meaning seems to be a separate step in the process of understanding other people’s intentions, indicating that the semantic nature of a proposition is a task on its own, relatively independent of speech acts (Hagoort and Indefrey, 2014, p. 359).

## Summary and conclusion

The purpose of this study has been to review a range of existing empirical publications that use semantic algorithms to predict and model psychological variables and their relationships. We argue that the nature and pervasiveness of semantic predictability should draw attention to how nomological networks can be re-interpreted as semantic networks. Further, we argue that the human capability for processing semantic networks might itself be an important psychological research object, characterized by the following features:

1. It provides a rule-oriented predictability to people’s behavior unlike any other behaviors except biological features of the nervous system. This predictability is probably an overlooked, strong law of psychology.
2. It is a measurable structure on an abstract mathematical level that seems to pervade all languages. This feature of the semantic grid provides a measure of similarity in meaning allowing us to translate expressions within and between languages. This points to a biological foundation of the semantic grid that enables culture. It is an open question how much the semantic grid is shaped by culture in return.
3. The computational character of the semantic grid is a constructive feature: It allows survey items and experimental variables to be grouped in accordance with theoretical definitions such that they can be turned into latent constructs. On the other hand, this function constitutes a large matrix that really makes all latent constructs related in some way or other, just like no concepts can exist in isolation from a semantic network. Thus, the “nomological network” argued by Cronbach & Meehl might be determined by (or even be identical to) processing in the semantic grid.
4. One feature of the semantic grid is its automatized character that hides it from conscious experience and hence from psychological investigation. This has led psychological research to adopt a canon for construct validation that locks it in an explanatory room limited upwards to around 42%. Research questions above this threshold will be regarded as same-construct questions. Conversely, for research questions to be argued, they will usually build on semantic networks existing in the semantic grid, driving the *a priori* relationships upwards. The resulting human blindness towards *a priori* relationships is a valid topic for psychological research on its own.
5. The semantic grid does not map truth values. It can only map the mutual meaning of concepts and statements, also for totally fictitious or erroneous ones. It is however sensitive to nonsense.
6. The semantic grid functions as the general matrix within which all definitions and measurement issues take place, forming our epistemic foundations in psychology and creating the “psychological manifest image.” We need to recognize and describe it to move past it.
7. The semantic grid is the key standardized communication platform for intersubjective mapping of reality across people. It can be modelled mathematically across subjective experiences as the REC-MTMM model.

## Practical implications

The current state of natural language processing allows researchers to assess how respondents are congruent with the semantic grid. The methodological possibilities are only starting to emerge. For example, it can be used in survey research as follows: At the item level, semantic similarity provides an objective measure that could be used as support for correlating errors in structural equation models. At the scale level, semantic similarity can be used to assess to what degree, if any, empirical nomological networks are based simply on the semantic similarity between item sets (Nimon and Shuck, 2020). In the instrument development stage, semantic similarity can be useful in developing items that are similar to the construct to be measured and divergent from other measures (Rosenbusch et al., 2020; Nimon, 2021). Rather than using eye-ball tests of semantic similarity, researchers can use increasingly available NLP tools to quantify the semantic similarity between two or more item sets or even for automated content validation (Larsen et al., 2023), allowing researchers to quantify discriminant validity.

Studying individual semantic behavior opens the door for future research. In prior research (Arnulf et al., 2018a,b,c,d, 2020; Arnulf and Furnham, 2024), individual semantic acuity or compliance has been shown to be related to personality and cognitive ability. Semantic acuity measures may also be useful in as control variables or to assessing common method variance in lieu of a marker variable, as well as to “unbundle the sample” (Bernardi, 1994, p. 772), identifying subgroups of individuals who yield differential item functioning based on their semantic behavior.

On a more epistemic level, we believe that conceptualizing the semantic grid and its computational properties can help psychology advance to better distinguish between semantically determined and empirically determined discoveries. Semantic computations might be used as a Bayesian priors for separating semantic from empirical relationships.

The rapid development of computerized text analysis and production will probably make text computations as prevalent in the field as factor analysis has been for the recent decades (Arnulf et al., 2021). We believe that psychology can adopt and adapt such tools to make more fruitful distinctions between semantic and empirical questions in the future.

## Limitations

This has been a review of already published studies that use semantic algorithms to predict empirically obtained data patterns. While these studies have found to be predictive of up to around 90% of the observed variation, the claim here is not that all data are semantically determined, nor that the semantic predictions may predict the observed data accurately. The claim is instead that with these possibilities of *a priori* predictions, the nature and meaning of empirical data needs to be considered in light of what is semantically predictable.

Contextual, cultural and statistical factors will always influence the relationships between semantic representations and their observed, empirical counterparts. These influences may be important objects of investigation or disturbing noise depending on the research questions at hand.

Finally, this article has not attempted to make an exhaustive description of how semantic calculations work as it would go beyond the present format. The specific algorithms used and the way the models are designed will affect how the features of the statistics are captured (Arnulf et al., 2018a,b,c,d). However, all reviewed studies contain published descriptions of the technology used. Natural language processing is rapidly advancing at the time of writing, rendering previously published methods less interesting in the future.

## Author contributions

JA: Conceptualization, Writing – original draft. UO: Writing – original draft, Writing – review & editing. KN: Writing – original draft, Writing – review & editing.
